# Access to medicines for rare diseases: A European regulatory roadmap for academia

**DOI:** 10.3389/fphar.2023.1142351

**Published:** 2023-02-28

**Authors:** Noa Rosenberg, Sibren van den Berg, Nina N. Stolwijk, Bart A. W. Jacobs, Hendrika C. Post, Anna M. G. Pasmooij, Saco J. de Visser, Carla E. M. Hollak

**Affiliations:** ^1^ Medicine for Society, Platform at Amsterdam UMC—University of Amsterdam, Amsterdam, Netherlands; ^2^ Expertise Center for Inborn Errors of Metabolism, Department of Endocrinology and Metabolism, Amsterdam UMC, Amsterdam Gastroenterology Endocrinology Metabolism (AGEM) Research Institute, MetabERN, University of Amsterdam, Amsterdam, Netherlands; ^3^ Department of Pharmacy, Amsterdam UMC—University of Amsterdam, Amsterdam, Netherlands; ^4^ Department of Pharmacy and Pharmacology, The Netherlands Cancer Institute—Antoni van Leeuwenhoek, Amsterdam, Netherlands; ^5^ Department of Oncology, Amsterdam UMC, University of Amsterdam, Amsterdam, Netherlands; ^6^ Dutch Medicines Evaluation Board, Utrecht, Netherlands; ^7^ Center for Blistering Diseases, European Reference Network-Skin Reference Center (ERN-Skin), University Medical Center Groningen, University of Groningen, Groningen, Netherlands; ^8^ Centre for Future Affordable & Sustainable Therapy Development (FAST), The Hague, Netherlands

**Keywords:** regulatory framework, patient access, marketing authorization, alternative drug-to-market routes, rare diseases, orphan drugs

## Abstract

**Background**: Novel or repurposed medicines for rare diseases often emerge from fundamental research or empirical findings in academia. However, researchers may be insufficiently aware of the possibilities and requirements to bring novel medicinal treatment options to the patient. This paper aims to provide an easily applicable, comprehensive roadmap designed for academic researchers to make medicines for rare diseases available for patients by addressing the relevant regulatory frameworks, including marketing authorization and alternative routes.

**Methods**: Key points of the regulatory chapters “Placing on the Market” and “Scope” of Directive 2001/83/EC relating to medicinal products for human use were summarized. Provisions in EU directives regarding blood products, radiopharmaceuticals, and herbal and homeopathic medicinal products were excluded. Cross-referencing to other provisions was included. European case-law was retrieved from the InfoCuria database to exemplify the implications of alternative routes.

**Results**: Medicines may only be placed on the market with a valid marketing authorization. To obtain such authorization in Europe, a “Common Technical Document” comprising reports on quality and non-clinical and clinical studies must be submitted to a “competent authority”, a national medicine agency or the European Medicines Agency. Timely interaction of academic researchers with regulators *via* scientific advice may lead to better regulatory alignment and subsequently a higher chance for approval of academic inventions. Furthermore, reimbursement by national payers could be essential to ensure patient access. Apart from the marketing authorization route, we identified multiple alternative routes to provide (early) access. These include off-label use, named-patient basis, compassionate use, pharmacy compounding, and hospital exemption for Advanced Therapy Medicinal Products.

**Discussion**: Aligning academic (non-)clinical studies on rare diseases with regulatory and reimbursement requirements may facilitate fast and affordable access. Several alternative routes exist to provide (early) pharmaceutical care at a national level, but case-law demonstrates that alternative routes should be interpreted strictly and for exceptional situations only. Academics should be aware of these routes and their requirements to improve access to medicines for rare diseases.

## 1 Introduction

In the Europe, access to medicines can be particularly challenging for patients with rare diseases, (Deticek et al., 2018). Since 2000, the European Commission (EC) has implemented policy to implement incentives for the development of medicines targeting these diseases *via* so-called orphan designations (European Medicines Agency, 2022a). One of these incentives is the 10-year market exclusivity upon authorization as an orphan medicinal product. To qualify for an orphan designation, the following criteria must be met (European Medicines Agency, 2022a):

1. Prevalence in the European Union (EU) is < 1:2,000
2. The disease is life-threatening or seriously debilitating
3. There are no satisfactory methods or—in case there is a satisfactory method—the product must be of significant benefit

Up until 2021, more than 200 orphan medicinal products, have entered the market in the EU (European Medicines Agency, 2020a). However, with 5,000 to 10,000 rare diseases (Haendel et al., 2020), many patients are left without pharmaceutical care.

New or repurposed medicines often emerge from fundamental research or empirical findings in academia (Oprea et al., 2011; van den Berg et al., 2021), but frequently do not reach the patient. This could be explained by misalignment with regulatory requirements (van den Berg et al., 2021), negative reimbursement decisions (Deticek et al., 2018), or lack of commercial interest. Academics could become more engaged along the drug development chain in an effort to improve this. For instance, academia-driven public-private partnerships could be explored that include social principles such as cost-saving and socially responsible pricing. Such collaborations might improve academic inventions reaching the market (Organisation for Economic Co-operation and Development, 2008; Padhy and Gupta, 2011; Nederlandse Federatie van Universitair Medische Centra, 2019; Pushpakom et al., 2019) or ameliorate complex and lengthy reimbursement negotiations that may result from initial prices exceeding national reimbursement thresholds, often in combination with the inevitable paucity of effectiveness data (Vella Bonanno et al., 2017; Deticek et al., 2018).

In striving for improvement of patient access to scientific inventions, academics should be acquainted with the drug development process and the associated regulatory aspects. Currently, knowledge of regulatory routes to patient access is frequently insufficient (Kallio et al., 2022) and technology transfer offices that support academics in expediting drug development generally focus on patenting and out-licensing (Van Norman and Eisenkot, 2017a; Van Norman and Eisenkot, 2017b). Therefore, improving awareness and education on ways to bring medicinal inventions to the patient and the corresponding regulatory framework is important (STARS, 2022).

The regulation of medicinal products started approximately a century ago and implementation has largely been driven by tragedies such as sulfanilamide (Can Med Assoc J., 1937; Western Journal of Medicine, 1938), thalidomide (Kim and Scialli, 2011), etc., understandably adding regulations thereby creating one of the most regulated industries focusing roughly on four parameters:

• Quality: Are the product characteristics consistent over time and consistent with the product for which safety and efficacy was demonstrated?
• Safety: When used as intended, are the potential side effects and/or risks acceptable?
• Efficacy: How well does the medicinal product achieve its intended clinical effect?
• Pharmacovigilance: What are the adverse effects when the authorized medicine is used in regular healthcare practice?

The regulations are traditionally focused on the industry applying for marketing authorization, commonly referred to as FDA- or EC-approval. However, interactions between academics and regulators are expanding and of particular value in rare diseases (Ruperto, 2005; European Medicines Agency, 2017). At present, the pharmaceutical legislation is under revision by the EC, which might create new opportunities for acadamics (European Commission, 2021). As academics frequently find novel interventions for these diseases with typically large unmet needs, they are increasingly participating in public private partnerships with the aim of obtaining market approval. Additionally, regulators can benefit from concentrated expertise in academic institutes for advice on regulatory decision-making.

So far, regulatory education for academics has been limited. The EMA has several resources to foster development from early phases in academic laboratories (European Medicines Agency, 2020b). Besides, multiple tools have emerged to support academic drug developers, such as the IRDiRC Orphan Drug Development Guidebook (Hechtelt Jonker et al., 2020), the Market Approval Navigator (Paul Janssen Futurelab Leiden, 2022a), and the educational Horizon 2020 project “Strengthening Training of Academia in Regulatory Science (STARS)” (Starokozhko et al., 2021). Several publications describe opportunities for academic researchers (Davies et al., 2017; Begley et al., 2021), including regulatory procedures, timelines, and fees to obtain an orphan designation and marketing authorization (Davies et al., 2017). These tools can assist academics in obtaining a marketing authorization, but do not encompass the overall helicopter view towards patient access.

This paper aims to provide an easily applicable, comprehensive roadmap designed for academic researchers to make medicines for rare diseases available for patients by addressing the relevant regulatory framework including marketing authorization and alternative medicine-to-patient routes.

## 2 Methods

The regulatory framework is composed of routes to make medicines for rare diseases available for patients through 1) marketing authorization route and 2) alternative medicine-to-patient routes.

### 2.1 Marketing authorization

Key points are summarized of the regulatory chapter “Placing on the Market” of the Directive 2001/83/EC of the European Parliament and of the Council of 6 November 2001 on the Community code relating to medicinal products for human use (Directive). This Directive is the core legislative act of medicines in the EU and is publicly accessible *via* the EudraLex collection (European Commission, 2015). Provisions relating to medicinal products were selected, excluding those regarding blood products, radiopharmaceuticals, and herbal and homeopathic medicinal products. Cross-referencing to other provisions was included as well.

### 2.2 Alternative routes

Key points are summarized of the chapter “Scope” of the Directive 2001/83/EC. Provisions were included or excluded on the same criteria as described for the marketing authorization route. To better understand the interpretation of the alternative routes within the regulatory framework, we chose to describe purposively sampled case-law (judicial decisions) that exemplify the practical implications of the Directive. The InfoCuria database was used to retrieve judgments of the European Court of Justice (ECJ) in English that refer to the included provisions (InfoCuria Case Law, 2022).

The application of the legal framework and case-law for the academic healthcare professional are summarized as “implications.” To further illuminate complex regulatory matters we provided several illustrative examples of the described routes.

## 3 Results and implications

**FIGURE 1:**
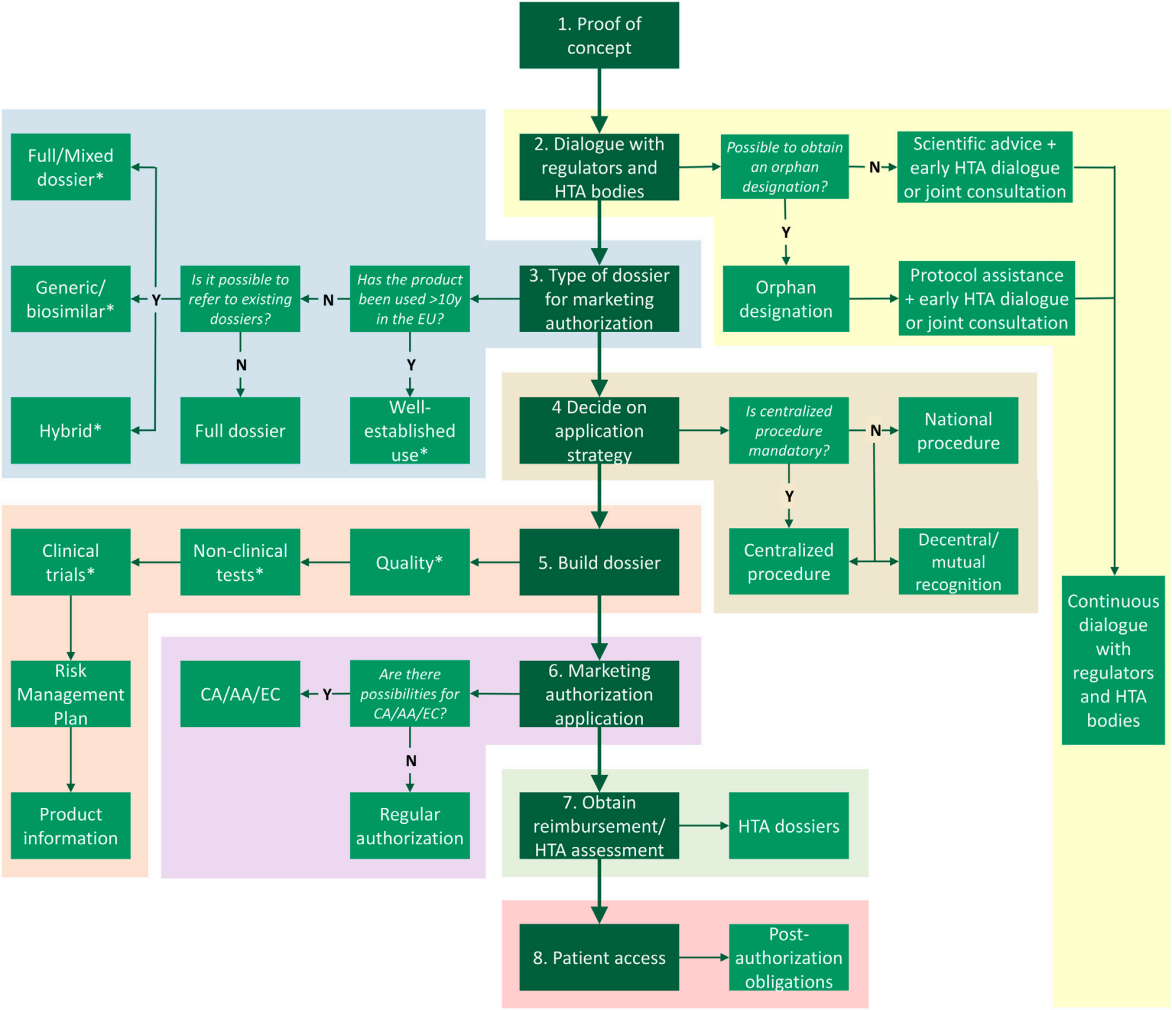
Route to patient access *via* marketing authorization. Following proof of concept there are multiple steps, including (1) dialogue with stakeholders, deciding what (2) type of dossier and (3) application procedure are suitable, (4) building the Common Technical Dossier, (5) submitting the marketing authorization application, (6) applying for the Health Technology Assessment (HTA) at a national HTA body, and (7) complying to post-authorization obligations such as pharmacovigilance. *Exemptions for certain (elements of) modules of the Common Technical Dossier may be applicable for authorized medicines. Y, yes; N, no; HTA, health technology assessment; CA, conditional approval; AA, accelerated assessment; EC, exceptional circumstances.

**FIGURE 2:**
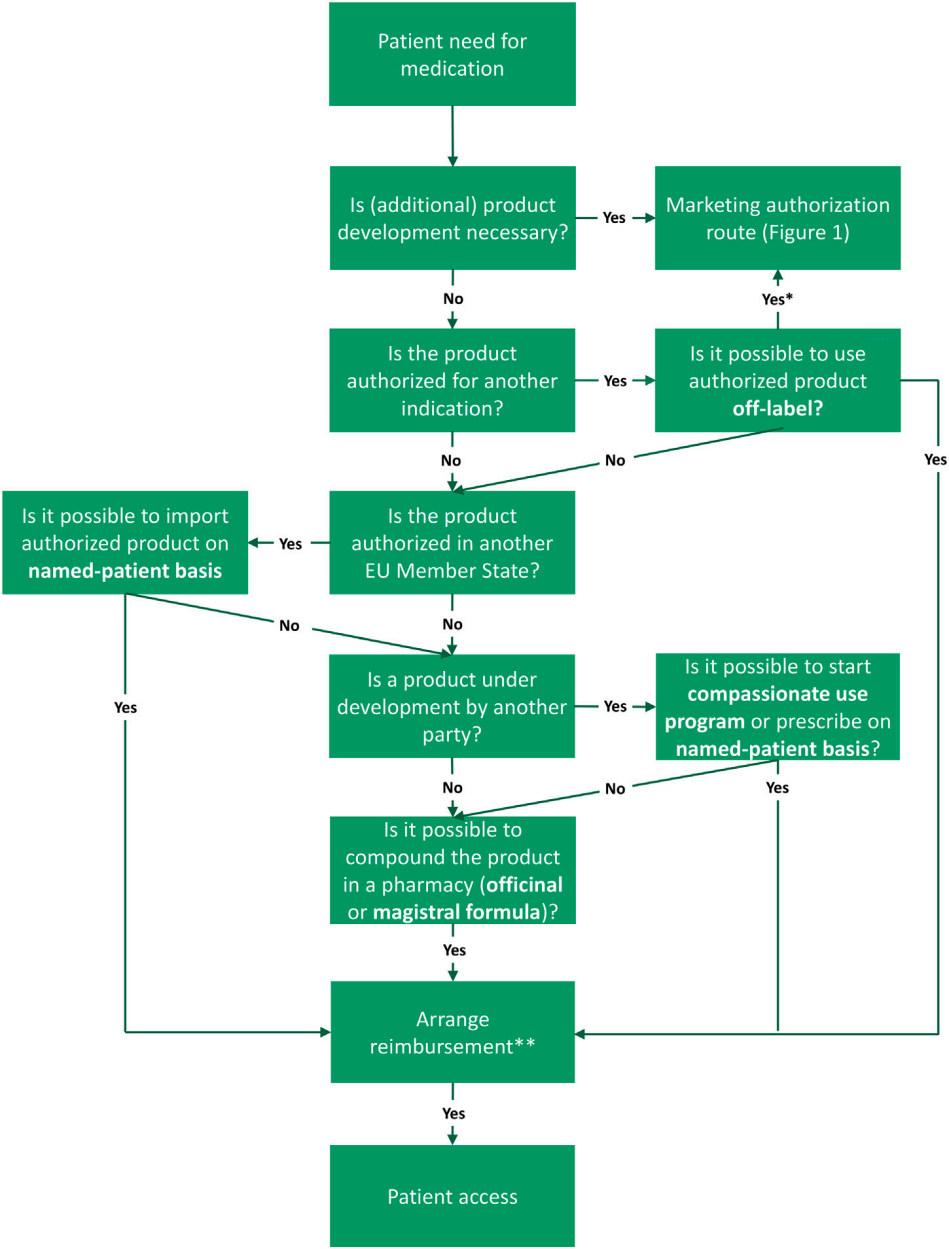
Routes to access following the need for a medicine in the European Union (EU), including alternative routes. There are multiple (decision) steps from unmet need to patient access (green boxes). Names of the routes are indicated in bold font. First step is that, in case of a patient need for medication, the academic should examine if product development is necessary, for example, because the product consists of a new active substance or a new formulation. When product development is needed, one should proceed with the marketing authorization route (see Figure 1). For products already authorized for another indication, off-label prescription is a viable option. However, the marketing authorization route can still be exploited to bring this indication on-label. If the product is authorized in another EU Member State, import on a named-patient basis could be suitable. In case the product is still under development by another party, it might be relevant to explore compassionate use programs or ways to prescribe an investigational product on named-patient basis. If these option are unattainable, pharmacy compounding *via* the magistral or officinal formula can offer a solution. In all cases, national reimbursement should be arranged in order to provide patient access. *Ideally, the marketing authorization route is executed to bring off-label uses on-label. **Reimbursement may following automatically, dependent on national legislation. Frequently, products facilitated *via* compassionate use programs are free of charge.

In this chapter, we outline the steps toward patient access following an academic invention through the marketing authorization route (Figure 1). Next, we will explain alternative medicine-to-patient routes with the aim to reaching access to both academic inventions, as well as to medicinal products developed by commercial parties (third parties) (Figure 2).

### 3.1 Marketing authorization

The most important rules regarding medicines in the EU are stipulated in Directive 2001/83/EC of the European Parliament and of the Council of 6 November 2001 on the Community code relating to medicinal products for human use (from here footnotes refer to legal provisions). This Directive asserts that medicinal products may only be placed on the market with a valid marketing authorization^1^. According to the Directive, a medicinal product is defined as a substance—or a combination of substances—presented as having properties for treating or preventing a disease^2^ and applies when “prepared industrially”^3^. Steps to obtain marketing authorization and subsequent access to the authorized medicine will be explained in the following paragraphs.

#### 3.1.1 Dossier

The first step towards marketing authorization is composing a dossier called “Common Technical Document” (CTD) which must be submitted to the European Medicines Agency (EMA) or a “competent authority”^4^ ^–^ ^6^, a national organization that is responsible for the authorization of medicines. This CTD consists of five modules, namely 1) administrative information, 2) summaries and overviews, 3) quality, 4) non-clinical study reports, and 5) clinical study reports^7^. The publicly available “CTD explained” animation clarifies the modules in the CTD and the compulsory steps to fill in this document (Paul Janssen Futurelab Leiden, 2022b).

1. Administrative information: The administrative information includes the orphan designation status, risk management plan, labeling, package leaflet, and required information about experts involved in the critical points related to quality, animal, and human investigations^8^.
2. The summaries and overviews: The summaries and overviews contain information on the quality of the product, and non-clinical and clinical aspects^9^.
3. The quality module: The quality module consists of all manufacturing details of the active substance and the finished product^10^. The difference between the active substance and the finished product is that the active substance(s) is the ingredient with pharmacological, immunological, or metabolic activity^11^ and the finished product is the dosage form, including all excipients and packaging material, which are required for marketing (e.g., a paper box with blisters that contain capsules)^12^.
4. The non-clinical study reports: The non-clinical study reports include pharmacological, pharmacokinetic and various toxicology studies^13^ ^,^ ^14^.
5. The clinical study reports: The clinical study reports contain pharmacokinetics, pharmacodynamics, and efficacy and safety studies^14^. In general, clinical trials should be performed as blinded randomized controlled clinical trials^15^. However, “exceptional circumstances” may be applicable for products that have an orphan designation and for which it is deemed impossible to provide complete (non-)clinical information^16^. This may be the case due to the rarity of a disease.

##### 3.1.1.1 Implications for academia

Results of academic (non-)clinical studies might not only be relevant for scientific publications, but could also be part of a Common Technical Dossier. When expediting this, studies should be performed according to Good Manufacturing Practice (GMP), Good Laboratory Practice (GLP) and/or Good Clinical Practice (GCP) (European Medicines Agency, 2022b; European Medicines Agency, 2023a; European Medicines Agency, 2023b). By adhering to these mandatory international minimum quality standards for manufacturing, non-clinical and clinical aspects, academics can demonstrate that the product they investigate is consistent and reliable. To ensure that studies comply to further regulatory requirements, academics should consider relevant long-term (non-)clinical endpoints and gain scientific advice or protocol assistance from regulatory authorities at an early stage. Implementation of regulatory advice may increase the chance of obtaining marketing approval (Hofer et al., 2015), avoid unnecessary additional studies, and expand incentives for further development, investments, and potential partnerships. Cost reductions on advice for academia may be applicable at a national level (STARS, 2022) and is free for products with an orphan designation at the EMA (European Medicines Agency, 2020c). Thus, obtaining an orphan designation could be a strategic first step for academics researching rare diseases (Davies, 2017). To do so, they have to submit an application at the EMA, generally containing scientific documentation on the medical condition, prevalence, other methods for diagnosis, prevention or treatment of the condition, and a description of the stage of development (European Medicines Agency, 2022c).

#### 3.1.2 Types of dossiers

The regulatory framework defines several legal bases for constructing the Common Technical Dossier. These legal bases describe various types of dossiers, of which some could be applicable to already authorized products and may exempt (a part of) studies^17^ ^,^ ^18^:

• Full dossier (Article 8(3)): Novel active substances require compiling a full dossier that consists of all above-mentioned modules.
• Full-mixed (Article 8(3)) or hybrid dossier (Article 10(3)): For known, repurposed, medicines used in a new indication or another application (e.g., method of administration), it is possible to constitute a full-mixed or hybrid dossier. In these cases, the dossier is a combination of studies and references to the original dossier^19^.
• Type II variation application: Current marketing authorization holders can submit a variation application to add a novel indication to the label. The application could be based on (non-)clinical studies, as well as bibliographic references^20^ (Commission Regulation (EC) No 1234/2008, 2008).
• Well-established use (Article 10a): Well-established use medicines are medicines that have been used in the EU for at least 10 years and for which efficacy and safety have been well established. This could be the case when a medicine has been used off-label. For this dossier, (non-)clinical tests and trials must be replaced by scientific literature^21^. In addition, for products with an orphan designation, systematic and documented use on a named-patient basis (see paragraph “Named-patient basis”) may be used in the dossier^16^.
• Generic medicines (Article 10(1)): Generic medicines are products with the same active substance and are bioequivalent to the original medicinal product. By proving this *via* a bioequivalency study, (non-)clinical studies can be replaced by references to the original dossier, but only after 8 years of data exclusivity on the original dossier have expired^17^. As long as this period has not expired, the applicant may only refer to the original dossier with the informed consent (Article 10c) of the current marketing authorization holder^22^ ^,^ ^23^.
• Biosimilars (Article 10(4)): Biosimilars are biological products that are highly similar to an approved biological medicine. Similar to generics, references to (non-)clinical studies^24^ may be made to the original dossier after the data exclusivity period. Contrary to generics, however, additional (non-)clinical studies are frequently necessary to prove the similarity in quality of the product^25^.

##### 3.1.2.1 Implications for academia

Academic researchers should be aware of the existence of various types of dossiers and necessary studies. As academics are frequently involved in repurposing (Austin and Gadhia, 2017; Begley et al., 2021; Roessler et al., 2021), they may have a particular interest in full (Box 1), full-mixed and well-established use dossiers (Box 2). Academics could obtain assistance from a competent authority and investigate what type of data is necessary to complement references to an original dossier. The prevention of replicates of studies and ownership of additional data may be a cost-saving strategy and thus contribute to affordable medicines. Besides (non-)clinical study related elements, academics are up to date on scientific literature and commonly perform systematic reviews. These endeavors may not only be scientifically relevant, but also for drafting a dossier for medicines used off-label based on well-established use.

BOX 1 An illustrative example of academic endeavors in obtaining a full dossier. Researcher A has been researching the pathology of a type of cancer for several years and identified a novel target. Structure libraries showed that the active substance in Medicine 1 might have a strong affinity to this target. Medicine 1 is available as low-dosage tablets and a different dosage and a new formulation are necessary for the oncological application. Researcher A finds a GMP-certified partner for formulation, manufacturing and quality aspects. Next, Researcher A consults the EMA on quality aspects and study design in (non-)clinical studies to ensure that the outcomes can be used for a Common Technical Dossier.

BOX 2 An illustrative example of academic endeavors in obtaining a well-established use dossier. Medicine 2 has been used off-label for decades. Its use has been well-documented in scientific literature and is incorporated in international guidelines. The off-label indication is not mentioned in the package leaflet, which leads to frequent questions from patients. Physician B wishes to build a well-established use dossier containing a systematic review of (non-)clinical research. Physician B applies for scientific advice from the EMA on whether such well-established use dossier is adequate and if so what outcome variables should be collected in the systematic review. By incorporating EMA’s advice, Physician B finalizes a protocol for this review that will make up an important part of the Common Technical Document. Concerning the quality and manufacturing elements, Physician B collaborates with a GMP-certified contracted manufacturing organization.

#### 3.1.3 Routes for application of the dossier

Once the dossier has been drafted, an application must be submitted^5^. There are four types of procedures to apply for marketing authorization, namely the centralized, national, decentralized, and mutual recognition procedures^26^.

• Centralized procedure: The centralized procedure refers to an application at the EMA which will lead to marketing authorization in all EU Member States^27^. This is mandatory for several types of medicines, including products with an orphan designation, medicines for oncological indications, biologicals, and cell- and gene therapy products^28–31^.
• National procedure: For the national procedure, the applicant will submit the dossier to a competent authority which will lead to marketing authorization in that Member State only.
• Decentralized and mutual recognition procedures: These procedures allow marketing authorization applications in multiple Member States concurrently or consecutively^29^.

##### 3.1.3.1 Implications for academia

As the centralized route is mandatory for products with an orphan designation, the EMA may be the most relevant authority^30^ for academics that perform research on rare diseases. National authorities may provide additional support for academics and can be an initial point of contact (STARS, 2022).

#### 3.1.4 During the assessment of the dossier: Accelerated access

When a marketing authorization application has been made, the concerned competent authority (or authorities) will assess the dossier on substantiated quality and the benefit-risk balance. There may be several routes on the European and national level to provide accelerated access^31,^ ^32,^ in case of unmet medical need, for instance, accelerated assessment and conditional approval.

• Accelerated assessment: The accelerated assessment speeds up the reviewing process from 210 to 150 days. A request can only be submitted for products with a major public interest.
• Conditional approval: The conditional approval grants authorization based on less comprehensive clinical data than normally required. After additional obligations have been met, the conditional approval can be switched into a “regular” marketing authorization^33–38^.
• Exceptional circumstances: Exceptional circumstances can apply when it is deemed impossible to obtain comprehensive data on efficacy and safety due to rarity of the disease or ethical considerations^34^.

##### 3.1.4.1 Implications for academia

When researchers are involved in the development of a medicine relating to a rare disease with an unmet need, they should be aware of the possibilities to initiate the previously mentioned options and apply for scientific advice as early as possible (particularly regarding exceptional circumstances) or other schemes that offer support during development, such as PRIME. This is a scheme that provides early and proactive support to optimize robust data generation and prepare for accelerated assessment (European Medicines Agency, 2022d; European Medicines Agency, 2022e).

#### 3.1.5 From authorization to national reimbursement

Once an application is assessed and a marketing authorization is granted, the product may be placed on the market in the respective Member States^35,^ ^36^ and the marketing authorization holder should continue monitoring the safety of the medicine according to Good Pharmacovigilance Practice (GVP) (European Medicines Agency, 2022f) . Although the centralized procedure grants authorization in all EU Member States, there is no obligation to actually market adult medicines in every Member State. Furthermore, the individual Member States are responsible for national healthcare reimbursement schemes and may include or exclude the medicine from these schemes^37^. In general, national public organizations called Health Technology Assessment (HTA) bodies will scrutinize the evidence for effectiveness and cost-effectiveness as well as appropriate use, before recommending or deciding on reimbursement (Vreman et al., 2020; Schoot et al., 2022). To facilitate (fast) patient access, several initiatives for collaboration have been set up between regulators and HTA bodies, such as the parallel joint scientific consultation between the EMA and the European Network of Health Technology Assessment (EUnetHTA) (European Medicines Agency, 2022g).

##### 3.1.5.1 Implications for academia

Early dialogue with HTA bodies and payers may be essential for academics, the industry^38,^ ^39^, and patients to expedite routes to reimbursement. Investigator-initiated research on efficacy, safety and effectiveness could be aligned with requirements from competent authorities and HTA bodies to obtain both marketing authorization and reimbursement. To harmonize this, academics could retrieve joint scientific advice. Obligations might be imposed regarding post-marketing data generation on safety, pregnancy and/or effectiveness. These data could be gathered *via* independent international registries (Jonker et al., 2017). Moreover, academics could be involved in the reimbursement assessment procedure of commercial products of third parties^40,^ ^41^. They can embrace a larger role by acting as clinical experts in committees involved in reimbursement recommendations or by proactively discussing appropriate use of the new product in clinical setting, but should be wary of potential conflicts of interest.

#### 3.1.6 Data ownership and protection

Apart from obtaining marketing authorization, acquiring ownership of data and intellectual property is an important step in product development. The industry typically strives to obtain (licenses to) patents, data exclusivity, and/or market protection (KNAW, 2021), because this allows a company time to generate sufficient sales to recoup its investment by restricting other companies from entering the market. These tools could also be advantageous for academia in valorization and finding partners for non-academic aspects, such as manufacturing and distribution. Several options are included in the regulatory framework:

• Patents: Patents can be granted to novel technical inventions with an industrial application^42^. When medicines are protected by patents^17^ ^,^ ^20^ ^,^ ^43^ ^,^ ^44^, the patent holder earns the right to prevent others from commercially exploiting the invention for at least 20 years^45^. However, necessary clinical trials with the intention of applying for marketing authorization are exempt from this protection^44^. The publicly available patent portfolio calculator provides a fairly accurate estimate of all costs related to the filing of a patent application, the granting procedures and patent maintenance in a large number of countries around the world (Paul Janssen Futurelab Leiden, 2022c). Once received, the patent can be assigned or licensed to another party (European Patent Office, 2023). Frequently this is in return for royalties: a percentage of the revenues generated by the company exploiting the patented invention (European Patent Office, 2022).
• Data exclusivity: For novel authorized products data exclusivity of 8 years applies, which restricts references to an approved dossier (by generic or full-mixed dossiers)^17^.
• Market protection: market protection applies for up to 10 years, with the possibility to obtain a 1-year extension if a new indication is added to the marketing authorization within the first 8 years^17^. This means that a generic medicinal product may refer to the originator dossier after 8 years (at the end of data exclusivity), but may be placed on the market only after the marketing protection period has passed.
• Market exclusivity: For orphan medicinal products, a market exclusivity of up to 10 years will be active for particular authorized indication(s), regardless of patent protection. This means that it is, in principle, prohibited to market a similar medicinal product for the same indication(s)^46–47^.

##### 3.1.6.1 Implications for academia

Academics may be insufficiently aware of the importance of the abovementioned protective tools and incentives. When aiming to bring treatments to patients, academics could consider ownership and protection of their data and intellectual property before publishing with the help of their technology transfer offices that are typically established to support academic researchers in the patenting process. When an institute has protected an invention, its (commercial) value increases. This could, in turn, lead to a lower risk and larger assurance of return on investments and gives the academic a stronger position for potential collaboration with industrial parties, for instance to set requirements on eventual patient access (KNAW, 2021). The same is true when academic institutes are prepared to co-invest in the development of a new medicinal product, i.e. by participating in clinical trials at a reduced cost rate, or to maintain disease registries that can be used to collect natural history^47^ data, safety and efficacy outcomes, and post-authorization evidence (Hollak et al., 2020). These independent registries are more cost-effective and have led to more completeness than industry-funded registries (Sirrs et al., 2021).

### 3.2 Alternative routes

Although a marketing authorization is mandatory to place a medicinal product on the market^40^, there are several provisions and exemptions in Directive 2001/83/EC that leave room for alternative medicine-to-patient routes. These alternative medicine-to-patient routes may provide both access to unauthorized products and/or uses following academic inventions, as well as access to commercial products from third parties (Figure 2). The following alternative routes will be discussed: off-label use, named-patient basis, compassionate use, magistral and officinal formulas, and the hospital exemption. The referenced case-law illustrates the interpretation of Directive 2001/83/EC (Supplementary Material S1).

#### 3.2.1 Off-label

Off-label use is the use of an authorized medicinal product that is not in accordance with the Summary of Product Characteristics, such as different indication, dosage, duration of use or patient group (Nivel et al., 2017). As EU legislation does not cover off-label use, it is not prohibited to use a medicine for unauthorized indications (F. Hoffmann-La Roche Ltd and others v Autoritá Garante della Concorrenza e del Mercato, 2018), national legislation may apply and regulate off-label prescription (Nivel et al., 2017; Caminada et al., 2021).

##### 3.2.1.1 Implications for academia

Off-label use may be a route to provide patients with a commercially available product after positive results from academic repurposing studies or initial empirical clinical findings (Box 3). Clinical practice shows that off-label use is common—for instance for pediatric uses—but varies in terms of underlying scientific substantiation (Nivel et al., 2017). National legislation may be in place, stipulating prerequisites for off-label prescription (Nivel et al., 2017; Caminada et al., 2021). In France, physicians are allowed to prescribe off-label medicines although they must justify their choice and inform the patient (Nivel et al., 2017). In Italy, there are several specific requirements, such as unmet need, support of completed phase II study and patient consent (Nivel et al., 2017). Legislation may also be in place for reimbursement of off-label use. In Netherlands, for example, the effectiveness of the product is a prominent element for reimbursement decisions (Nivel et al., 2017). As there is a large heterogeneity of off-label legislation between Member States (Caminada et al., 2021), national requirements should be scrutinized before academic repurposed inventions can be lawfully prescribed to patients. The possible shortcoming of evidence and unharmonized legislation may stress the need for bringing new off-label indications on-label, for instance by aligning investigator-initiated repurposing studies with regulatory and HTA requirements.

BOX 3 An illustrative example of off-label use. Researcher C performed research on the pathology of a rare metabolic disease and found a potential drug target. To further investigate this in the laboratory, researcher C uses the active substance from an authorized medicine that is known for inhibiting this target. Following positive results, C inquires joint scientific advice from regulators and HTA bodies to initiate a multi-center pivotal trial. This trial leads to positive results and the use of the medicine gets adopted in international clinical guidelines. As a consequence, medical specialists start to prescribe this already commercially available medicine off-label. In some Member States, the product will be reimbursed automatically (Nivel et al., 2017). In other Member States, a (case-by-case) application and assessment have to be performed (Nivel et al., 2017).

#### 3.2.2 Named-patient basis

For individual patients with special needs, a medicinal product can be provided on a “named-patient basis”. This means that Member States may allow unapproved medicines that are prescribed by a doctor based on therapeutic considerations to fulfill pharmaceutical needs^48^, when there are no authorized equivalents available (European Commission v Republic of Poland, 2012; Abcur AB v Apoteket Farmaci AB and Apoteket AB, 2015). This exception can be used to facilitate access in various ways. First, this route could be used for access to medicinal products that are still under development following academic inventions. Second, commercial investigational products could be supplied on a named-patient basis, for example, when a marketing authorization application in currently under review. Third, the named-patient basis can permit import (European Commission v Republic of Poland, 2012; F. Hoffmann-La Roche Ltd and others v Autoritá Garante della Concorrenza e del Mercato, 2018a) of medicines that are commercially available in other countries but do not have a marketing authorization in the country where the patient is treated. However, named-patient basis must remain exceptional and cannot be used to avoid using authorized products or omit obtaining marketing authorization for financial reasons (Supplementary Material S1) (European Commission v Republic of Poland, 2012; F. Hoffmann-La Roche Ltd and others v Autoritá Garante della Concorrenza e del Mercato, 2018a). Last, data gathered through the systematic and documented use of named-patient basis may be part of a well-established use dossier for orphan medicines^16^.

##### 3.2.2.1 Implications for academia

Due to the individual character of the named-patient procedure, this can enable access to investigational products or products^49^ only available in another country for the treatment of patients with rare diseases or very small patient subsets in exceptional situations (Box 4). For this procedure, EU Member States impose various obligations, such as approval of prescriptions and physician’s statements, import permits, and pharmacovigilance (Kreeftmeijer-Vegter et al., 2013). If this is the intention to use data obtained on named-patient basis for a marketing authorization^16^, protocol assistance should be obtained from regulators on data collection to compose a positive benefit/risk balance. More guidance from regulators might be essential to improve use of aggregated named patient data for regulatory purposes. This could help academics bring medicines to patients and avoid unnecessary trials.

BOX 4 An illustrative example of named-patient import. Physician D prescribes immediate-release pills to his patients with a neuromuscular disorder. One of his patients does not respond well to the immediate-release pills. Physician D would like to investigate whether modified-release pills would be more suitable. Physician D finds out that modified-release pills are only authorized in EU Member States with a higher disease prevalence. Physician D contacts the company and urges to apply for a marketing authorization. Unfortunately, the company is not interested in this because of the extremely small patient population. To obtain the modified-release pills, Physician D inquires about local requirements for import on a named-patient basis at the pharmacy, national competent authority, health inspectorate and the insurance company of the patient. Physician D learns that forms need to be submitted at the health inspectorate and the insurance company that substantiate rational use of the product. Physician D provides sufficient scientific evidence to arrange the import of the product and the reimbursement for the individual patient.

#### 3.2.3 Compassionate use

Besides prescription of investigational products on named-patient basis, compassionate use is a way to provide access to an unauthorized medicine for a cohort of patients with a life-threatening or seriously debilitating disease who cannot be treated with an authorized therapy. To facilitate such program, the sponsor has to initiate this procedure with a competent authority when clinical trials are ongoing or when a marketing authorization application has been made^49^.

##### 3.2.3.1 Implications for academia

When engaged in pre-marketing studies, physicians can stimulate manufacturers to apply for compassionate use programs to supply the product during or after the trials until the authorized medicine becomes readily available (Box 5). Steps to comply with these obligations should be taken in advance to make sure patients have continuous access. Furthermore, data collected *via* these programs could be used for regulatory filings and HTA assessments (Polak et al., 2020; Polak et al., 2022).

BOX 5 Illustrative example of compassionate use. Medicine 3 is under development for a rare neurodegenerative disorder with an unmet need. The sponsor is currently executing the final clinical trial and aims to apply for market approval within 1 year. One of the inclusion criteria of this trial is to be able to perform a 6 min walk test. Therefore, several seriously ill wheelchair bound patients are ineligible. Their treating physician has been involved in the trial and contacts the sponsor with a request to provide access to Medicine 3 for these patients. The sponsor applies for a compassionate use program at the national competent authority. Following a positive decision, the sponsor will supply Medicine 3 for the wheelchair bound patients *via* compassionate use.

#### 3.2.4 Magistral and officinal formulation

An alternative route to access is *via* pharmacy compounding. This is the preparation of medicinal product in a pharmacy (Abcur AB v Apoteket Farmaci AB and Apoteket AB, 2015). There are two types of pharmacy compounding that are exempt in Directive 2001/83/EC, namely the magistral formula and the officinal formula^50,^ ^51^:

• Magistral formula: the magistral formula is a medicine that is prepared according to a medical prescription for an individual patient^50^. This means magistral formulas may only be compounded for a specific patient after a physical examination of that patient and issuing the prescription (Abcur AB v Apoteket Farmaci AB and Apoteket AB, 2015).
• Officinal formula: the officinal formula is a medicine that is prepared in accordance with the pharmacopeia (the quality reference work of medicines) and is intended to be supplied to the patients served by the preparing pharmacy in question^51^. Case-law clarified that officinal formulas may only be delivered directly to patients of the pharmacy that prepared them and not to another pharmacy’s patients (Abcur AB v Apoteket Farmaci AB and Apoteket AB, 2015).

Pharmacy compounding *via* the magistral or officinal formula thus provides local access routes to unauthorized products or applications. This can be applicable to academic pharmaceutical inventions or to products that are similar to medicinal products authorized by third parties (Abcur AB v Apoteket Farmaci AB and Apoteket AB, 2015), for instance to overcome a temporary shortage.

##### 3.2.4.1 Implications for academia

Often pharmacy preparations are compounded for individual patients with therapeutical needs, for example, a suitable formulation or dosage for a pediatric patient. Because of its individual character, the magistral formula forms an interesting local route for rare diseases with very few patients (Box 6). As the officinal formulas lack the individual element, they can be compounded before a prescription has been issued on (small) stock (Box 7) (Bruins, 2019). This makes the officinal formula more applicable to emergency settings or larger groups of patients. To be able to compound it is understandable that the active substance needs to be available and formulation should not violate patents, though many EU Member States exempt pharmacy preparations for individual cases from infringement (Visser, 2010).

BOX 6 An Illustrative example of a magistral formulation. There are only a few patients with a rare cancer in the European Union. An investigator-initiated trial with a compounded product showed promising results. The trajectory to access *via* the marketing authorization route takes years. In the meantime, Physician E wants to prescribe this product to his patient. Based on this prescription, the local pharmacy prepares the product *via* a magistral formulation. Reimbursement decisions will vary. In Belgium, for example, E needs to write a report to confirm the diagnosis (Rijksinstituut voor ziekte-en invaliditeitsverzekering, 2022a; Rijksinstituut voor ziekte-en invaliditeitsverzekering, 2022b). In Netherlands, rational pharmacology needs to be demonstrated to the healthcare insurer. This includes that the medicinal product needs to be a suitable pharmaceutical formulation, has proven efficacy and effectiveness, and is the most economical option (Ministerie van Volksgezondheid Welzijn en Sport, 2022a).

BOX 7 An illustrative example of an officinal formulation. Medicine 4 was occasionally used off-label for a rare disease. For financial reasons, the marketing authorization holder decided to withdraw the medicine from the market. A pharmacy was notified that several patients would be left without treatment and started the preparation in accordance with the pharmacopeia, as an officinal formulation, for its patients.

#### 3.2.5 Hospital exemption for ATMPs

For advanced therapy medicinal products (ATMPs), which are gene therapy medicinal products, somatic cell medicinal products, or tissue-engineered products^52^, there is the possibility of a “hospital exemption.” In the case of an individual prescription (as discussed above), custom-made ATMPs may be exempt from marketing authorization^53^. These medicines are prepared on a non-routine basis according to specific quality standards and these medicines should be used in a hospital under the exclusive professional responsibility of a medical practitioner, within the same Member State as where they are manufactured^53^. The manufacturing needs to be authorized by national competent authorities and/or inspectorates (Coppens et al., 2020).

##### 3.2.5.1 Implications

For academics, the hospital exemption provides a controlled local route for tailor-made ATMPs, such as personalized gene therapy for patients with very rare genetic disorders (Box 8). Besides, for seriously debilitating and life-threatening diseases, this may provide a route to commercially developed products from third parties that are not authorized yet. However, with varying national requirements on quality, efficacy and safety and subsequent concerns on potential public health impact (Coppens et al., 2020; Hills et al., 2020), the hospital exemption route should only be pursued with the utmost care.

BOX 8 An illustrative example of a hospital exemption. Clinician-scientist F discovered a new genetic defect causing a rare disease. In collaboration with other scientists, F developed a gene therapy product and commenced pre-clinical research which shows promising results. In good coordination with regulators, natural history data from a disease register can serve as a comparator for the clinical study that F is planning. In case the study demonstrates positive outcomes, F aims to provide access to the treatment as soon as possible. Apart from gathering advice from regulators on marketing authorization, he obtains information on the possibility to produce the tailor-made gene therapy under the hospital exemption and inquires about necessary documentation for relevant national authorities. To guarantee the data’s suitability for future marketing authorization application, F will ensure that the product is manufactured at a GMP-certified facility and that the studies and trial comply with GLP and GCP standards.

#### 3.2.6 Alternative routes and reimbursement

There are multiple alternative medicine-to-patient routes in the European regulatory framework, to which varying national rules apply, particularly regarding reimbursement (Rigter et al., 2021). Hence, it is imperative that the relevant national rules should be reviewed and, if applicable, corresponding organizations should be engaged from the onset to ensure patient access, e.g. through joint scientific advice. The differences between reimbursements of alternative medicine-to-patient routes can be exemplified by the Dutch system. Medicines used off-label are usually reimbursed automatically, (Zorginstituut Nederland, 2022) apart from medicines to which specific prescription restrictions apply. (Zorginstituut Nederland, 2022; Ministerie van Volksgezondheid Welzijn en Sport, 2022b). For other routes, several conditions must be met, i.e., that there are no authorized adequate alternative medicines available and that use of the product is rational pharmacology (Zorginstituut Nederland, 2022). Nonetheless, imported medicines for rare diseases that affect less than < 1:150,000 inhabitants are exempt from these conditions and will generally be compensated for.

## 4 Actionable recommendations

Based on the regulatory framework and relevant implications discussed, the following actionable recommendations can be set forth for the marketing authorization route and alternative medicine-patient routes.

Marketing authorization:

• Academics researching medicinal inventions for rare diseases should align research and clinical practice with regulatory requirements with the aim of obtaining marketing authorization and subsequent reimbursement to ensure long-term patient access. This potentiality can be bolstered when cooperating with technology transfer offices, industry, and patients.
• Academics should actively connect with regulators and HTA bodies to successfully align their research, for example, by applying for an orphan designation and/or scientific advice. This will also improve academia’s value in potential partnerships.
• Academics should not shy away from a more prominent position in drug development and become aware that this could support social terms regarding eventual access in collaborations.
• Universities/academic medical centers should establish regulatory offices or integrate knowledge on regulatory advice within existing Technology Transfer Offices and Centers of Entrepreneurship that help researchers navigate through the labyrinthine regulatory landscape.

Alternative routes:

• Clinicians should contact their pharmacists and relevant stakeholders such as healthcare insurance companies to discuss alternative routes and arrange coverage if necessary for local patient access.
• Academics could examine whether efficacy, safety and/or effectiveness data collected *via* alternative routes can serve the purpose of marketing approval and/or reimbursement.

## 5 Discussion

This regulatory roadmap is the first published guidance for academics to facilitate access to medicines for rare diseases. This framework indicates that there are several routes to patient access, both by developing an authorized medicinal product as well as *via* alternative medicine-to-patient routes, such as off-label or named-patient use. An authorized product offers the highest assurance of quality, efficacy, and safety and is the preferred option. To obtain marketing authorization for an academic invention, product development is necessary and a comprehensive dossier needs to be compiled and submitted to a competent authority (Davies et al., 2017). While this process requires extensive regulatory knowledge, often present in industry, academics should be aware that they can educate themselves and consequently expand their position. For instance, they can inquire scientific advice or protocol assistance, either central through the EMA or at a national level (STARS, 2022). Aligning investigator-initiated (non-)clinical studies with requirements for marketing authorizations and reimbursement could be crucial to determine end-points, improve chance of marketing approval and prevent access delays (Hofer et al., 2015; Ofori-Asenso et al., 2020). Besides streamlining research with formal requirements, academics could approach drug development trends proactively by considering establishing disease registries, patenting inventions, and data sharing (Van Norman and Eisenkot, 2017a; Van Norman and Eisenkot, 2017b; Sirrs et al., 2021; Karpen et al., 2021; Jansen-van der Weide et al., 2018; Jones et al., 2011; Schoenmakers et al., 2022). To support these effort, academic institutes could improve “in-house” regulatory knowledge to guide researchers through the regulatory roadmap.

In general, clinicians have alternative routes close-at-hand and can reach patient access through these alternatives typically together with their pharmacist. Case-law demonstrated, however, that these exceptional routes should be interpreted with caution and should not be executed because of financial interests. Furthermore, when proceeding with an alternative route, reassuring quality, safety and efficacy for patients should still be priority, similar to the essence of the regular framework. For example, scrutinizing strategies on how to utilize the resultant data for marketing authorization or reimbursement might be an activity more eminently dedicated to academics. The subsequent expanded position of academia in the drug development process could empower a more prominent stand in collaboration with regulators, HTA-bodies, and industry. This might even inforce an improved negotiation position on socially responsible terms when, for example, affordable pricing of an invention is what is deemed important from the academic perspective.

The current European legislation is built on the pharmaceutical industry that traditionally aimed for developing medicines for large patient populations and relatively recently consolidated incentives to develop medicines for rare diseases. Nowadays, we face a new “innovation wave” comprising of ATMPs and more personalized medicines (Konar et al., 2022). Medicinal products for rare diseases are a frontrunner in this paradigm shift from larger general indications to tailored personalized approaches, but are often authorized with less evidence on effectiveness evoking complicated reimbursement negotiations and impeded patient access (Malinowski et al., 2018). A modernization to adapt to the latest practice should also apply to the regulatory framework from authorization to reimbursement including bridging the gap between efficacy (authorization) and effectiveness in daily practice (reimbursement). The EU pharmaceutical legislation is currently under revision (European Commission, 2021)—of which the Clinical Trials Regulation entered into force beginning of 2022 (European Commision, 2014; European Medicines Agency, 2022h)—and will hopefully harmonize these components. Including possibilities to integrate alternative routes and marketing authorization routes in the EU legislation might help aligning academic endeavors and clinical practice with marketing authorization. The arena of (ultra-)rare diseases is an evident first step to do so, due to the (extremely) small patient populations, concentrated clinical expertise, and control of expenditures. Further research on the coalescence of routes, together with regulators, patients, and industry, is essential to sculpture future patient-centered legislation.

## Footnotes

1 Directive 2001/83/EC art 6 para 1

2 Directive 2001/83/EC art 1 para 2(a)

3 Directive 2001/83/EC art 2 para 1

4 Directive 2001/83/EC art 6 para 1

5 Directive 2001/83/EC art 8 para 1

6 Directive 2001/83/EC annex I para 1

7 Directive 2001/83/EC annex I para 2

8 Directive 2001/83/EC annex I part I-1

9 Directive 2001/83/EC annex I part I-2

10 Directive 2001/83/EC annex I part I-3

11 Directive 2001/83/EC art 3a

12 Directive 2001/83/EC Annex I part I art 3.2.2.1

13 Directive 2001/83/EC annex I part I-4

14 Directive 2001/83/EC annex I part I-5

15 Directive 2001/83/EC annex I part I-5.2.5.1

16 Directive 2001/83/EC Annex I Part III art 5

17 Directive 2001/83/EC art 10 para 1

18 Directive 2001/83/EC art 10 para 2(b)

19 Directive 2001/83/EC arts 10 para 4

20 Directive 2001/83/EC art 10a

21 Directive 2001/83/EC art 10c

22 Directive 2001/83/EC art 8(3)

23 Directive 2001/83/EC art 10 para 3

24 Directive 2001/83/EC art 10

25 Directive 2001/83/EC annex I part II-4

26 Directive 2001/83/EC art 28

27 Directive 2001/83/EC annex I para 3

28 Regulation (EC) No 726/2004 annex I para 1

29 Regulation (EC) No 726/2004 annex I para 1a

30 Regulation (EC) No 726/2004 annex I para 3

31 Regulation (EC) No 726/2004 annex I para 4

32 Directive 2001/83/EC art 26 para 1

33 Regulation (EC) No 726/2004 art 14 para 9

34 Regulation (EC) No 726/2004 art 14-a

35 Commission Regulation (EC) No 507/2006 art 4

36 Commission Regulation (EC) No 507/2006 art 5

37 Commission Regulation (EC) No 507/2006 art 6

38 Commission Regulation (EC) No 507/2006 art 7

39 Regulation (EC) No 726/2004 art 14(8)

40 Directive 2001/83/EC art 6

41 Regulation (EC) No 726/2004 art 3 para 1

42 Directive 2001/83/EC art 4 para 3

43 The European Patent Convention art 52

44 Directive 2001/83/EC art 10 para 6

45 Regulation (EC) No 141/2000 art 8 para 1

46 The European Patent Convention art 63 para 1

47 Regulation (EC) No 141/2000 art 8 para 2

48 Directive 2001/83/EC art 5 para 1

49 Regulation (EC) No 726/2004 art 83

50 Directive 2001/83/EC art 3 para 1

51 Directive 2001/83/EC art 3 para 2

52 Regulation (EC) No 1394/2007 art 2 para 1(a)

53 Directive 2001/83/EC art 3 para 7
