# Supplementary material for: Access to medicines for rare diseases: A European regulatory roadmap for academia
Source: Front Pharmacol. 2023 Feb 28;14:1142351. doi: 10.3389/fphar.2023.1142351 (PMC10012277; doi:10.3389/fphar.2023.1142351)
Supplement: Supplementary file 1 [file Table1.DOCX]

Supplementary Material

Access to medicines for rare diseases: a European regulatory roadmap for academia

Noa Rosenberg MSc^1,2^, Sibren van den Berg MSc^1,2^, Nina N. Stolwijk MD^1,2^, Hendrika C. Post MD^1,3^, Bart A.W. Jacobs PharmD PhD^1,3,4^, Anna M.G. Pasmooij PhD^5,6^, Saco J. de Visser PhD^1,7^, Carla E.M. Hollak MD PhD^1,2^*

*** Correspondence:** Carla E.M. Hollak MD PhD: [c.e.hollak@amsterdamumc.nl](mailto:c.e.hollak@amsterdamumc.nl)

# Supplementary Table

**Supplement 1. Summaries of statements regarding routes in the costs or consideration in English Judgements from the European Court of Judgement.**

| CASE nUMBER | Parties | Year | Summary |
| --- | --- | --- | --- |
| C-29/17 | Novartis Farma SpA v Agenzia Italiana del Farmaco (AIFA) and Others | 2018 | **Magistral or officinal preparation outside pharmacy**: In this case, the medicine was not produced in dispensing or hospital pharmacies, but industrially in Roche’s laboratories, which holds its marketing authorization. This cannot be a seen as magistral or officinal preparation **Repackaging as magistral or officinal formula**: Repackaging processes of Avastin cannot be regarded as the ‘preparation’ of a new medicinal product as a magistral or officinal formula. |
| C-179/16 | F. Hoffmann-La Roche Ltd and others v Autorità Garante della Concorrenza e del Mercato | 2018 | **Off-label use**: The Directive 2001/83 does not prohibit the use of medicinal products for therapeutic indications not covered by their MA. |
| C-185/10 | European Commission v. Republic of Poland | 2012 | **Importing non-approved medicines**: Importing non-approved medicinal products must remain exceptional in order to preserve the practical effect of the marketing authorization procedure **Financial considerations**: Financial considerations cannot lead to recognition of the existence of special needs. The named patient procedure is not concerned with the organization of the national health-care system or its financial stability. The named patient procedure cannot be used to avoid a marketing authorization for financial reasons |
| C-276/15 | Hecht-Pharma GmbH v Hohenzollern Apotheke, Winfried Ertelt | 2016 | **100 packages in the course of one day**: Normal pharmacy manufacturing steps that produce up to 100 packages in the course of one day under pharmacy operating license for dispensation and supply cannot be regarded ~~‘~~prepared industrially’ or ‘manufactured by a method involving an industrial process’. This can be an officinal formula. |
| C-544/13 | Abcur AB v Apoteket Farmaci AB and Apoteket AB | 2015 | **Marketing authorization irrelevant for pharmacy preparation**: Magistral formulas can only be prepared in accordance with a medical prescription issued before their preparation, which must be for a previously identified patient. Officinal formulation can only be delivered directly to patients supplied by the pharmacy which prepared them. For these preparations it is irrelevant whether there are other authorized medicines with the same active substance, same dosage and same pharmaceutical form.  **Standardized stock, wholesale, large-scale magistral formula**: Standardized stock production and wholesale selling and the large-scale or serial production of magistral formulae in batches are characteristic of ‘industrial preparation’ or ‘manufacture by a method involving an industrial process’. This cannot be a magistral formula.  **Named patient procedure only when no authorized equivalent**: The named patient procedure can only concern situations in which the doctor considers that his patient’s state of health requires a medicine for which there is no authorized equivalent available on the national market. When there is an authorized equivalent available, there cannot in fact be a question of ‘special needs’ for a named patient procedure. It should be interpreted strictly, applicable in exceptional cases where it is appropriate to meet special medical needs. |
